# Current Approaches to Neuromodulation in Primary Headaches: Focus on Vagal Nerve and Sphenopalatine Ganglion Stimulation

**DOI:** 10.1007/s11916-016-0577-5

**Published:** 2016-06-08

**Authors:** Francesca Puledda, Peter J. Goadsby

**Affiliations:** NIHR-Wellcome Trust King’s Clinical Research Facility, King’s College London, London, UK; Department of Neurology and Psychiatry, Sapienza University of Rome, Rome, Italy; Wellcome Foundation Building, King’s College Hospital, London, UK

**Keywords:** Neuromodulation, Headache, Migraine, Cluster headache, TACs, Peripheral nerve stimulation, nVNS, SPG

## Abstract

Neuromodulation is a promising, novel approach for the treatment of primary headache disorders. Neuromodulation offers a new dimension in the treatment that is both easily reversible and tends to be very well tolerated. The autonomic nervous system is a logical target given the neurobiology of common primary headache disorders, such as migraine and the trigeminal autonomic cephalalgias (TACs). This article will review new encouraging results of studies from the most recent literature on neuromodulation as acute and preventive treatment in primary headache disorders, and cover some possible underlying mechanisms. We will especially focus on vagus nerve stimulation (VNS) and sphenopalatine ganglion (SPG) since they have targeted autonomic pathways that are cranial and can modulate relevant pathophysiological mechanisms. The initial data suggests these approaches will find an important role in headache disorder management going forward.

## Introduction

Headache disorders are the most common form of disability on a global basis and the sixth most common cause of disability worldwide [1••]. The cumulative lifetime incidence of migraine approaches 50 % in females if one includes probable migraine and chronic migraine [2], while the 1 year prevalence for cluster headache, for which there is no cumulative data, is about 0.1 % of the population [3, 4]. Although the majority of people affected by primary headache disorders can be classified as episodic, a percentage of patients develop chronic forms often resistant to regular pharmacological treatment, which result in an enormous burden for sufferers and difficulties for physicians. Estimated societal burdens run to billions in the USA [5] and Europe [6••]. On the other hand, even patients with less severe headache syndromes can develop noticeable side effects with medical therapy and therefore require a constant pursuit for new treatment options.

These growingly recognized problems have led to the expansion of an exciting new branch of headache treatment: neuromodulation. This group of techniques comprises non-invasive treatments which, by targeting the central or peripheral nervous system, aim at modifying pain and other mechanisms involved in headache, and more invasive surgical approaches directed towards structures directly involved in the genesis of specific headache syndromes. Here, we have been tasked to cover neuromodulation of autonomic pathways plausibly intersecting with migraine [7, 8••] and trigeminal autonomic cephalalgia (TAC, [9••]) neurobiology.

## Non-invasive Vagus Nerve Stimulation (nVNS)

### Background

VNS has been used as treatment for epilepsy [10, 11] and depression [12] for many years. Invasive devices have dominated use and are variably accepted in clinical practice. The vagus nerve is the tenth cranial nerve. It is a mixed sensory and motor nerve with a long course and many functions, which is paired. It shall be referred to in the singular unless laterality is relevant. It arises from or converges upon

- The dorsal motor nucleus of the vagus (DMNX): parasympathetic visceral efferents
- The nucleus ambiguous: parasympathetic cardiac preganglionic fibres
- The nucleus of the solitary tract (NTS): afferent taste and visceral afferents
- The spinal trigeminal nucleus: ear, posterior fossa dura and larynx.

It is considered to be substantially an afferent nerve in terms of identified fibres, with between 65 and 80 % being sensory, although this often cited figure is based on feline data [13]. Upon exiting the medulla oblongata, it descends and exits the cranium through the jugular foramen. The nerve conveys parasympathetic preganglionic fibres widely and returns limited cutaneous sensory and widespread visceral afferent traffic. It is notable that the right vagal nerve innervates the sinoatrial node to slow the heart rate, while the left vagus innervates the atrioventricular node.

#### VNS and Trigeminal Pain

A bundle of afferent fibres of the vagus nerve, along with the glossopharyngeal and facial nerve fibres innervating the ear and the larynx, terminates in the trigeminal nucleus caudalis [14]. Furthermore, the nucleus tractus solitarius—the main nucleus of the vagus nerve—has shown to receive dural nociceptive afferents [15, 16]. Physiological studies demonstrated an effect of vagal afferents on non-cranial nociceptive pathways [17–19]. Vagal stimulation can modulate the pial blood flow [20]. However, acute vagotomy in experimental animals does not alter craniovascular responses due to sphenopalatine ganglion activation [21]. Most recent studies in rats demonstrate that vagus nerve stimulation can reduce pain and allodynia [22, 23] in the trigeminal basin. This may be mediated by an ascending antinociceptive effect of the vagus nerve on the second order neurons of the spinothalamic and spinoreticular tract responsible for the spinal nociceptive transmission to the trigeminal nuclear complex [24, 25]. One suggested mechanism is a reduction in the glutamate levels and of neuronal firing in the spinal trigeminal nucleus secondary to continuous vagus stimulation [22]. Notably, no cardiac side effects were reported in any of the studies, even though they are theoretically possible based on the efferent projections of the nucleus ambiguous to the preganglionic parasympathetic cardiac neurons. This could be due to the pulse wave of vagal nerve stimulator devices that are specifically designed to preferentially activate A- and B-myelinated fibres and not parasympathetic C fibres of the vagus nerve [26, 27].

### Migraine

Initial convincing attention of a possible effect of VNS in patients came from an epileptic patient implanted with a VNS device, whose epilepsy was not responsive but that noted a reduction in migraine headache shortly after the beginning of the treatment [28]. Sometime later, VNS devices were implanted in patients with refractory headache without epilepsy with some limited success [29]. Similarly, two other seizure cohorts have shown improvements in migraine with implanted VNS, although with some change in seizure frequency [30, 31], thus making it difficult to infer causality in this case. Lastly, a useful effect on migraine has been reported in patients with VNS for depression who also had improvements in migraine [32]. These early reports provided some context for the clinical studies on a nVNS.

Recently, a portable transcutaneous non-invasive device that stimulates the cervical portion of the vagus nerve has been developed (GammaCore®), with animal studies demonstrating that its effects are similar to those of implanted stimulators [33]. The nVNS is administered by placing the device on the neck, which then produces a mild electrical current that is transmitted to the vagus nerve through the skin [34••]. The treatment has been used in primary headaches with very promising results and a high safety and tolerability rate.

#### Acute Attack Treatment with nVNS

The first large pilot study to investigate nVNS in migraine was an open-label single-arm trial aimed at treating acute attacks [35]. In this study, 27 patients with episodic migraine treated 80 attacks with two unilateral 90-s doses, separated by 15-min intervals. Of the 54 moderate or severe attacks, 22 % were completely aborted at 2 h, while 43 % had a significant reduction in pain scores. This effect is comparable to that of similarly tolerated triptans. Side effects were generally mild, infrequent and well tolerated; the ones more clearly associated with the treatment itself were neck twitching, raspy voice and redness over the application site on the neck. A further open-label study was conducted to treat headache worsenings in patients with chronic migraine [36]. Twenty-two patients, 18 females, treated 79 attacks, with ≥50 % reduction in VAS at 2 h in 46 % of patients. Another recent open-label, single-arm, multicenter study investigated the use of nVNS for the acute treatment of high-frequency episodic and chronic migraine. A total of 131 attacks were treated by 48 patients with two unilateral 120-s doses of nVNS at 3-min intervals. At 2 h, the pain-relief rate was 51.1 % and the pain-free rate was 22.9 %. The positive response to the device was more evident in the subgroup of patients with lower frequency of attacks [37].

#### Preventive Treatment with nVNS

Regarding prevention, initial results with VNS seem quite promising. In a small Belgian study, 18 patients—12 with migraine—were treated with transcutaneous VNS. In total, ten discontinued the treatment because of lack of efficacy and/or side effects, although one patient with medication overuse had a reduction in more than 50 % of headache frequency [38]. Silberstein and colleagues recently performed a double-blind, sham-controlled pilot study using nVNS as a preventive in chronic migraine. The treatment—two 90-s doses given three times a day—was performed in the blinded phase on 59 patients for 2 months and was followed by a 6-month open-label phase. At the end of the 2-month double-blind phase, there was a −1.9 day (*n* = 26) reduction in headache days in the active and a 0.2 day (*n* = 23) change in headache days in sham (*p* = 0.12) [39], leaving open a question of how long one should treat to achieve neuromodulation. Another randomized sham-controlled study for the prevention of episodic migraine is ongoing and currently recruiting patients (NCT02378844).

### Cluster Headache (CH)

Two CH patients were reported to benefit from an implanted vagus nerve stimulator [29]. Following the availability of nVNS and its increased use, an audit of 19 patients with active episodic (*n* = 8) or chronic (*n* = 11) CH was reviewed over a 12-month period [40•]. Patients were instructed to administer up to three consecutive doses for the acute treatment of an attack, whereas for the preventive use, they were to self-administer 2–3 consecutive doses twice a day. The treatments were given for 120 s unilaterally to the side of the headache; the intensity of the stimulation was controlled by the patient. Results were encouraging: 79 % of patients reported an overall improvement of their initial conditions of approximately 50 %. In these “responders”, around half of the attacks were aborted in less than 15 min, and the attack frequency was also reduced of nearly 50 % respect to baseline during the treatment period. No serious adverse events were reported, and the treatment showed good tolerability in most patients.

In a recent prospective multicenter randomized controlled trial, nVNS was compared to the standard of care in the preventive treatment of chronic cluster headache [41]. Data from the 93 patients included in the analysis showed CH attacks were significantly reduced of 7.6 per week with nVNS treatment. This consisted of three stimulations twice daily for preventive treatment, as well as optional acute treatments for attack rescue. Furthermore, patients used a lower amount of rescue medications and showed good safety and tolerability with nVNS. Recently, a multicenter, double-blind, randomized controlled study to evaluate the efficacy and safety of acute CH treatment with nVNS has been completed (NCT01792817), as well as a study on the acute treatment of episodic and chronic cluster headaches (NCT01958125). Results of these trials are eagerly awaited. Finally, one CCH patient from the Belgian cohort previously mentioned had a significant attack reduction with prophylactic tVNS. Two patients from the same group were diagnosed with hemicrania continua, one of which had an initial decrease in pain intensity with tVNS followed by a quick relapse; results are not available for the second patient [38].

## Sphenopalatine Ganglion (SPG) Stimulation

### Background

The sphenopalatine (pterygopalatine) ganglion [42, 43] is a major outflow pathway for the facial (VIIth) nerve cranial dilator system [44, 45], which is the efferent portion of the trigeminal-autonomic reflex [46]. This system arises from neurons in the superior salivatory nucleus [47] that receive inputs from trigeminal nucleus caudalis [48]. The SPG is a hexamethonium-sensitive nicotinic ganglion [21] containing vasoactive intestinal polypeptide (VIP) [49, 50], pituitary adenylate cyclase-activating peptide (PACAP) [51] and nitric oxide synthase [52]. This pathway is the basis for canonical cranial autonomic symptoms such as lacrimation, conjunctival injection, nasal symptoms, aural symptoms and periorbital oedema, when activated typically by trigeminal nociceptive afferents [53•]. Thus, experimentally induced pain [54], migraine [55, 56] and the trigeminal autonomic cephalalgias (TACs) [46] all share expression of the pathway, with a remarkable differentiation in prominence, reproducibility and lateralization of the symptoms in TACs [57].

Maizels [58, 59] demonstrated the effectiveness of nasal lidocaine-induced SPG block in reducing pain during migraine attacks. The first trial to attempt SPG stimulation for migraine treatment was a small pilot study performed by Tepper and colleagues [60], who applied electrical stimulation via a needle inserted in the sphenopalatine fossa through an infrazygomatic approach in 11 patients with refractory migraine. Induced attacks were aborted (*n* = 2) or relieved (*n* = 3) in only five patients, although the authors discuss that this relatively low response could be due to incorrect lead placement or concomitant medication overuse headache in most patients. At the moment, an RCT is testing the efficacy of an implanted microstimulator in migraine (NCT01540799), and another trial for the acute treatment of episodic migraine (NCT01294046) has been completed and not reported. A single case report of intractable facial pain presenting some migrainous features and treated with SPG modulation has also been published [61].

### Cluster Headache

Medically refractory cluster headache is a truly awful problem. Currently, such patients would be offered occipital nerve stimulation—ONS—[62, 63], although this approach still requires controlled evidence for its efficacy [64]. Patients have been offered deep-brain stimulation in the region of the posterior hypothalamic grey [65] based on earlier neuroimaging work [66]. Deep-brain stimulation failed its initial controlled trial [67] and has an established, albeit small, mortality [68], while ONS is disorder non-specific; better approaches are clearly needed.

Initial attempts at SPG manipulation involved ablation and nerve blocks, which were not particularly effective and carried the drawbacks of an invasive approach [69–72]. More recently, a small proof of concept study with five cluster headache patients examined the effect of percutaneous stimulation of the SPG in treating acute attacks, with positive results. The treatment, delivered through a removable electrode, caused a complete abortion in 11 of the 18 treated attacks as well as the resolution of cranial autonomic symptoms, when these were present [73].

Based on these encouraging findings, a miniaturized implantable neurostimulator was developed, containing a lead with six electrodes that is implanted in the pterygopalatine fossa close to the SPG and anchored to the zygomatic process of the maxilla [74••]. The device is controlled remotely by the physician or the patient, who can adjust the intensity based on the voltage at which deep paresthesias are evoked behind the root of the nose, indicating correct activation [75]. The efficacy and safety of this device were examined in a European-based randomized controlled trial in 28 refractory chronic cluster headache patients who received either active, sub-perception or sham stimulation [74••]. Of the 566 treated attacks, pain relief was achieved in 67 % of the attacks treated with full stimulation (of which the average frequency was 120 Hz) compared to 7 % in both sham and sub-threshold stimulation. Of the 28 patients, 19 (68 %) benefitted significantly from the treatment, with an effective acute attack response in 9 (32 %) as well as an unexpected frequency reduction in 12 (43 %). The most common side effects of treatment were sensory disturbances and pain secondary to the surgical implantation, which generally resolved completely. A long-term follow-up at 18 months is being completed, and preliminary data showed a sustained therapeutic benefit for 66 % of patients [76]. At the moment, a trial is enrolling to explore these positive results in the acute treatment of chronic CH (NCT02168764).

## Conclusions

The armamentarium for the treatment of migraine and the trigeminal autonomic cephalalgias is rapidly expanding thanks to neuromodulation techniques. The newer methods seem much better tolerated and offer important therapeutic benefits. Equally attractive in many ways is that bench-based understanding is being applied to neuromodulation to yield bedside advances in treatment. Clinicians can look forward to the results of a number of ongoing studies and the real possibility to add these exciting methods to their practice.
